# Bowel preparation for elective colorectal resection: multi-treatment machine learning analysis on 6241 cases from a prospective Italian cohort

**DOI:** 10.1007/s00384-024-04627-6

**Published:** 2024-04-16

**Authors:** Marco Catarci, Stefano Guadagni, Francesco Masedu, Giacomo Ruffo, Massimo Giuseppe Viola, Felice Borghi, Gianluca Garulli, Felice Pirozzi, Paolo Delrio, Raffaele De Luca, Gianandrea Baldazzi, Marco Scatizzi, The Italian ColoRectal Anastomotic Leakage (iCral) study group, Paolo Ciano, Michele Benedetti, Leonardo Antonio Montemurro, Marco Clementi, Elisa Bertocchi, Gaia Masini, Amedeo Altamura, Francesco Rubichi, Marco Migliore, Daniele Parlanti, Gabriele Vago, Antonio Sciuto, Ugo Pace, Andrea Fares Bucci, Michele Simone, Diletta Cassini, Lorenzo Pandolfini, Alessandro Falsetto, Ferdinando Ficari, Francesco Giudici, Fabio Cianchi, Alberto Patriti, Marcella Lodovica Ricci, Walter Siquini, Alessandro Cardinali, Stefano D’Ugo, Marcello Spampinato, Stefano Scabini, Alessandra Aprile, Domenico Soriero, Marco Caricato, Gabriella Teresa Capolupo, Giusto Pignata, Jacopo Andreuccetti, Ilaria Canfora, Andrea Liverani, Giuseppe Lamacchia, Claudia Franceschilli, Roberto Campagnacci, Angela Maurizi, Pierluigi Marini, Grazia Maria Attinà, Ugo Elmore, Francesco Puccetti, Francesco Corcione, Umberto Bracale, Roberto Peltrini, Roberto Santoro, Pietro Amodio, Massimo Carlini, Domenico Spoletini, Rosa Marcellinaro, Antonio Giuliani, Giovanni Del Vecchio, Mario Sorrentino, Massimo Stefanoni, Giovanni Ferrari, Pietro Maria Lombardi, Alberto Di Leo, Lorenzo Crepaz, Augusto Verzelli, Andrea Budassi, Giuseppe Sica, Giulia Bagaglini, Stefano Rausei, Silvia Tenconi, Davide Cavaliere, Leonardo Solaini, Giorgio Ercolani, Gian Luca Baiocchi, Sarah Molfino, Marco Milone, Giovanni Domenico De Palma, Giovanni Ciaccio, Paolo Locurto, Giovanni Domenico Tebala, Antonio Di Cintio, Luigi Boni, Elisa Cassinotti, Stefano Mancini, Andrea Sagnotta, Mario Guerrieri, Monica Ortenzi, Roberto Persiani, Alberto Biondi, Andrea Lucchi, Giulia Vitali, Dario Parini, Maurizio De Luca, Antonino Spinelli, Francesco Carrano, Michele Genna, Francesca Fior, Vincenzo Bottino, Antonio Ferronetti, Andrea Coratti, Giuseppe Giuliani, Roberto Benigni, Dario Scala, Battistino Puppio, Alessio Vagliasindi, Andrea Muratore, Patrizia Marsanic, Nicoletta Sveva Pipitone Federico, Maurizio Pavanello, Carlo Di Marco, Umberto Rivolta, Camillo Leonardo Bertoglio, Micaela Piccoli, Francesca Pecchini, Carlo Talarico, Vincenzo Greco, Alessandro Carrara, Michele Motter, Giuseppe Tirone, Mauro Totis, Nicolò Tamini, Franco Roviello, Riccardo Piagnerelli, Alessandro Anastasi, Giuseppe Canonico, Gianluca Guercioni, Simone Cicconi, Giuseppe Maria Ettorre, Marco Colasanti, Mauro Montuori, Enrico Pinotti, Pierpaolo Mariani, Roberta Carminati, Nicolò de Manzini, Edoardo Osenda, Annibale Donini, Luigina Graziosi, Mariano Fortunato Armellino, Ciro De Martino, Lucio Taglietti, Arianna Birindelli, Gabriele Anania, Matteo Chiozza, Mariantonietta Di Cosmo, Daniele Zigiotto, Carlo Vittorio Feo, Fioralba Pindozzi, Paolo Millo, Manuela Grivon, Corrado Pedrazzani, Cristian Conti, Silvio Guerriero, Lorenzo Organetti, Andrea Costanzi, Michela Monteleone, Nereo Vettoretto, Emanuele Botteri, Federico Marchesi, Giorgio Dalmonte, Massimo Basti, Diletta Frazzini, Graziano Longo, Simone Santoni, Moreno Cicetti, Gabriele La Gioia, Giuseppe Brisinda, Stefano Berti

**Affiliations:** 1 General Surgery Unit, Sandro Pertini Hospital, ASL Roma 2, Rome, Italy; 2 General Surgery Unit, Università degli Studi dell’Aquila, Via Vetoio, snc, 67100 L’Aquila, Italy; 3 Department of Biotechnological and Applied Clinical Sciences, Università degli Studi dell’Aquila, L’Aquila, Italy; 4 General Surgery Unit, IRCCS Sacro Cuore Don Calabria Hospital, Negrar di Valpolicella, Verona, VR Italy; 5 General Surgery Unit, Cardinale G. Panico Hospital, Tricase, LE Italy; 6 Oncologic Surgery Unit, Candiolo Cancer Institute, FPO-IRCCS, Candiolo, TO Italy; 7 General Surgery Unit, Infermi Hospital, Rimini, Italy; 8 General Surgery Unit, ASL Napoli2 , Nord, Pozzuoli, NA Italy; 9 Colorectal Surgical Oncology, Istituto Nazionale per lo Studio e la Cura dei Tumori, Fondazione Giovanni Pascale IRCCS-Italia”, Naples, Italy; 10 Department of Surgical Oncology, IRCCS Istituto Tumori “Giovanni Paolo II”, Bari, Italy; 11 General Surgery Unit, ASST Ovest Milanese, Legnano, MI Italy; 12 General Surgery Unit, Serristori Hospital, Santa Maria Annunziata &, Florence, Italy

**Keywords:** Colorectal surgery, Mechanical bowel preparation, Oral antibiotics, Anastomotic leakage, Surgical site infections

## Abstract

**Background:**

Current evidence concerning bowel preparation before elective colorectal surgery is still controversial. This study aimed to compare the incidence of anastomotic leakage (AL), surgical site infections (SSIs), and overall morbidity (any adverse event, OM) after elective colorectal surgery using four different types of bowel preparation.

**Methods:**

A prospective database gathered among 78 Italian surgical centers in two prospective studies, including 6241 patients who underwent elective colorectal resection with anastomosis for malignant or benign disease, was re-analyzed through a multi-treatment machine-learning model considering no bowel preparation (NBP; No. = 3742; 60.0%) as the reference treatment arm, compared to oral antibiotics alone (oA; No. = 406; 6.5%), mechanical bowel preparation alone (MBP; No. = 1486; 23.8%), or in combination with oAB (MoABP; No. = 607; 9.7%). Twenty covariates related to biometric data, surgical procedures, perioperative management, and hospital/center data potentially affecting outcomes were included and balanced into the model. The primary endpoints were AL, SSIs, and OM. All the results were reported as odds ratio (OR) with 95% confidence intervals (95% CI).

**Results:**

Compared to NBP, MBP showed significantly higher AL risk (OR 1.82; 95% CI 1.23–2.71; *p* = .003) and OM risk (OR 1.38; 95% CI 1.10–1.72; *p* = .005), no significant differences for all the endpoints were recorded in the oA group, whereas MoABP showed a significantly reduced SSI risk (OR 0.45; 95% CI 0.25–0.79; *p* = .008).

**Conclusions:**

MoABP significantly reduced the SSI risk after elective colorectal surgery, therefore representing a valid alternative to NBP.

**Supplementary Information:**

The online version contains supplementary material available at 10.1007/s00384-024-04627-6.

## Introduction

Current practice and recommendations regarding bowel preparation before elective colorectal surgery to reduce the incidence of anastomotic leakage (AL) and surgical site infections (SSIs) remain controversial. Mechanical bowel preparation (MBP), once routinely used, may cause preoperative dehydration, electrolyte disturbance, and discomfort, and failed to demonstrate any clear benefit over no bowel preparation (NBP) [1–5]. European [6] and Italian [7] enhanced recovery after surgery (ERAS) societies’ guidelines currently recommend NBP, albeit leaving room for oral antibiotics (oA) alone or in combination with MBP [8]. At the same time, results of large retrospective population-based studies of the American College of Surgeons National Surgical Quality Improvement Program (ACS-NSQIP) suggested that MBP combined with oral antibiotics (MoABP) significantly decreased the rates of SSIs and overall morbidity (OM) compared to NBP [9–13], inducing four large North-American societies (The American Society of Colon and Rectal Surgeons, the Society of American Gastrointestinal and Endoscopic Surgeons, the American Society for Enhanced Recovery, and the Perioperative Quality Initiative) to recommend MoABP [14–16]. As a consequence, the use of MoABP is currently reported by 50% of Austrian–German [17] and by 80% of North American [18] surgeons. During the last 8 years, one RCT was launched comparing NBP with MoABP [19], two MoABP with oA [20, 21], and one MoABP with MBP for rectal cancer [22]. To the best of our knowledge, only one [22] of these trials recently completed the planned enrollment and none published its final results yet [23]. An interesting four-arm RCT comparing NBP with oA, MBP, and MoABP for colon resections [24] was recently closed before completion due to poor accrual. Meanwhile, one RCT comparing NBP with MoABP [25] failed to detect significant differences in SSIs and AL rates but was largely underpowered; oA showed a significant reduction of SSI rates in two RCTs, either alone [26, 27] or combined with MBP [26], and an international multicenter RCT comparing oA with MoABP [28] is currently still recruiting. Finally, one RCT reported that MoABP significantly reduced SSI rates compared to MBP after colorectal resections [29], and another that MoABP significantly reduced both SSI and AL rates compared to MBP after rectal resections [30].

Very recently, the European Association of Endoscopic Surgery, the European Society of ColoProctology, and the Society of American Gastrointestinal and Endoscopic Surgeons published a joint guideline [31] based on a previous systematic review and network meta-analysis [32], with a conditional recommendation for MoABP, supported by low-quality evidence due to variable adherence to preoperative intravenous antibiotic prophylaxis (PIVAP) and great heterogeneity regarding oA schedules [33].

The relevant heterogeneity of the available evidence induced the Italian ColoRectal Anastomotic Leakage (iCral) study group to estimate the effects of NBP in patients treated with PIVAP before elective colorectal surgery (treatment variable) in comparison to three other treatments (oA, MBP, MoABP) on a large dataset derived from two prospective multicenter open-label observational studies [34, 35]. Several recent studies of propensity score estimation showed that machine learning methods outperform logistic regression models with iterative variable sections in terms of bias reduction and mean-squared error [36] and may be advantageous in multiple treatment settings [37]. Therefore, a multi-treatment analysis based on machine learning procedures was used to compare four bowel preparation modalities before elective colorectal surgery.

## Methods

### Study design, participants, and setting

This was a secondary unplanned ad hoc multi-treatment re-analysis of two prospective cohorts of patients who had undergone colorectal surgery for malignant and benign diseases based on machine-learning procedures. A total of 8359 patients who underwent colorectal resection with anastomosis were enrolled in two consecutive studies upon explicit inclusion/exclusion criteria in 78 surgical centers in Italy from January 2019 to September 2021: iCral2 [34] and iCral3 [35].

**Fig. 1:**
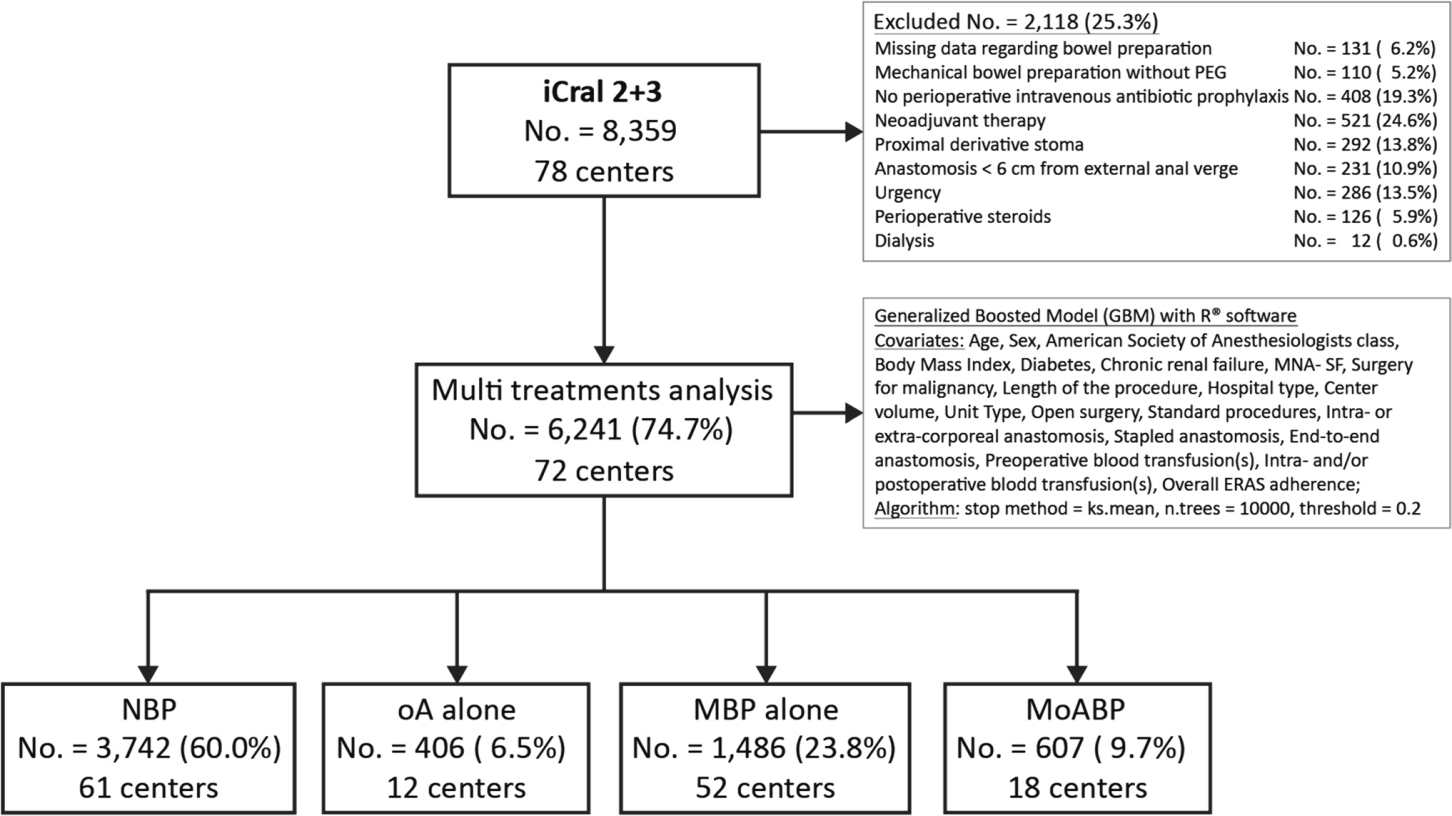
Study flowchart. PEG, polyethylene glycol; MNA-SF, mini nutritional assessment–short form [38]; ERAS, enhanced recovery after surgery; NBP, no bowel preparation; oA, oral antibiotics; MBP, mechanical bowel preparation; MoABP, mechanical bowel preparation and oral antibiotics

To control for data imbalance derived from several treatment confounders, the present analysis included 6241 patients (74.7%) out of 8359 available in the parent studies, based on explicit exclusion criteria (Fig. 1). Any record with missing information regarding preoperative bowel preparation or with MBP performed using anything different from polyethylene glycol (PEG) was excluded; patients treated without PIVAP were excluded considering its significant impact on the risk of SSIs [23]; delayed urgencies were excluded because this study is focused on elective resections; any anastomosis protected by a proximal stoma and patients treated with neo-adjuvant therapy, perioperative steroids, or dialysis were excluded because these treatments were impacting only on subgroups of subjects; patients treated by anterior resection with anastomosis at less than 6 cm from the anal verge and without protective stoma were excluded in relation to the significant impact of this procedure on the risk of AL. The study adhered to the Strengthening the Reporting of Observational Studies in Epidemiology statement [39] and checklist (online supplemental material).

Four different treatment groups were considered: (a) no mechanical bowel preparation and no oral antibiotics (NBP; No. = 3742; 60.0%); (b) oral antibiotics alone (oA; No. = 406; 6.5%); (c) mechanical bowel preparation alone (MBP; No. = 1486; 23.8%); (d) mechanical bowel preparation and oral antibiotics (MoABP; No. = 607; 9.7%). All patients in the MBP and MoABP groups received products containing PEG on the day before surgery. Patients in the oAB and MoABP groups received several different oral antibiotic schedules, the majority of which contained metronidazole (Table 1).

**Table 1:** Oral antibiotics schedules in the oA and MoABP groups

**Oral antibiotic(s)**	**Administration schedule**	**oA (406 pts.)**		**MoABP (607 pts.)**		p*
		No	%	No	%	
Metronidazole (500 mg) Paromomycin (250 mg)	Started 2 days preop., TID Started 2 days preop., BID	118	29.1	29	4.8	.006
Metronidazole (500 mg) Cefazolin (2000 mg)	Started 1 day preop., TID Started 1 day preop., OD	76	18.7	50	8.2	.102
Metronidazole (500 mg) Trimethoprim (160 mg)/sulfamethoxazole (800 mg)	Started 1 day preop., TID Started 1 day preop., TID	68	16.7	61	10.0	.267
Metronidazole (500 mg) Neomicin plus bacitracin (300 mg)	Started 1 day preop., TID Started 1 day preop., TID	47	11.6	6	0.9	.419
Metronidazole (500 mg) Amoxicilline (1000 mg)	Started 3 days preop., BID Started 3 days preop., BID	25	6.2	5	0.8	.623
Metronidazole (250 mg) Ciprofloxacin (500 mg)	Started 1 day preop., TID Started 1 day preop., BID	20	4.9	21	3.5	.823
Metronidazole (500 mg) Rifaximin (400 mg)	Started 7 days preop., TID Started 7 days preop., BID	5	1.2	9	1.5	.963
Metronidazole (250 mg) Amoxicilline (1000 mg)	Started 1 day preop., BID Started 1 day preop., BID	0	0	50	8.2	n.e
Metronidazole (250 mg) Rifaximin (200 mg)	Started 1 day preop., TID Started 1 day preop., BID	3	0.8	0	0	n.e
Metronidazole (500 mg) Rifaximin (200 mg)	Started 1 day preop., BID Started 1 day preop., BID	0	0	68	11.2	n.e
Metronidazole (1000 mg) Rifaximin (400 mg)	Started 1 day preop., TID Started 1 day preop., TID	0	0	11	1.8	n.e
Metronidazole (500 mg) Paromomycin (500 mg) Rifaximin (400 mg)	Started 1 day preop., BID Started 1 day preop., BID Started 1 day preop., BID	0	0	126	20.8	n.e
Paromomycin (250 mg)	Started 4 days preop., QID	44	10.8	0	0	n.e
Paromomycin (1000 mg)	Started 1 day preop., OD	0	0	37	6.1	n.e
Rifaximin (400 mg)	Started 1 day preop., TID	0	0	102	16.8	n.e
Amoxicillin (1000 mg)	Started 3 days preop., TID	0	0	17	2.8	n.e
Neomicin plus bacitracin (300 mg)	Started 1 day preop., TID	0	0	15	2.5	n.e

^*^OD once daily, *BID* 2 times per day, *TID* 3 times per day, (*QID*) 4 times per day, *preop*., preoperatively, *n.e*., test not executable because there are cells with insufficient values

^a^*t* test for proportions comparison, *oA* oral antibiotics

^b^*MoABP* mechanical bowel preparation plus oral antibiotics

### Clinical data

The parent studies recorded both continuous and discrete variables related to biometric data, patient information, indication and type of surgical procedure, adherence to ERAS program items, and outcomes. Local investigators ensured data quality control, which was validated by the study coordinator, resolving any discrepancies through strict cooperation. Perioperative care was provided by local investigators, who were left free to decide on any complimentary imaging and/or any further action according to local criteria.

The descriptive variables considered in the 6241 patients are shown in Table 2. Continuous variables were categorized according to their median values to optimize the effectiveness of the analysis by reducing the number of unmatched cases.

**Table 2:** Descriptive analysis of the variables considered in the 6241 patients before matching

		**NBP**		**oA**		**MBP**		**MoABP**		
		**No. = 3742**		**No. = 406**		**No. = 1486**		**No. = 607**		
**Variable**	**Pattern**	**No**	**%**	**No**	**%**	**No**	**%**	**No**	**%**	***p***
Age (years)	≤ 70	1863	49.8	203	50.0	882	59.4	342	56.3	< .001
	> 70	1879	50.2	203	50.0	604	40.6	265	43.7	
Sex	Male	1949	52.1	209	51.5	682	45.9	323	53.2	< .001
	Female	1793	47.9	197	48.5	804	54.1	284	46.8	
ASA class	I–II	2402	64.2	255	62.8	1.028	69.2	407	67.1	.003
	III	1340	35.8	151	37.2	458	30.8	200	32.9	
Body mass index (Kg/m^2^)	≤ 25.15	1803	48.2	234	57.6	765	51.5	323	53.2	< .001
	> 25.15	1939	51.8	172	42.4	721	48.5	284	46.8	
Diabetes	Yes	565	15.1	42	10.3	192	12.9	81	13.3	.020
	No	3177	84.9	364	89.7	1.294	87.1	526	86.7	
Chronic renal failure	Yes	154	4.1	18	4.4	65	4.4	27	4.4	.958
	No	3588	95.9	388	95.6	1.421	95.6	580	95.6	
MNA-SF	≤ 12	1971	52.7	166	40.9	883	59.4	309	50.9	< .001
	> 12	1771	47.3	240	59.1	603	40.1	298	49.1	
Surgery for malignancy	Yes	2713	72.5	312	76.8	992	66.8	427	70.3	< .001
	No	1029	27.5	94	23.2	494	33.2	180	29.7	
	Diverticular disease	535	52.0	60	63.8	142	28.7	107	59.4	
	Endometriosis	17	1.6	2	2.1	225	45.5	0	0.0	
	Polyps	214	20.8	18	19.1	47	9.5	17	9.5	
	IBD	142	13.8	6	6.4	16	3.3	22	12.2	
	Other	121	11.8	8	8.6	64	13.0	34	18.9	
Mini-invasive surgery	No	431	11.5	51	12.6	281	18.9	62	10.2	< .001
	Yes	3311	88.5	355	87.4	1.205	81.9	545	89.8	
	Laparoscopic	2790	84.2	317	89.3	1.006	83.5	509	93.4	
	Robotic	344	10.4	15	4.2	129	10.7	17	3.1	
	Converted	177	5.4	23	6.5	70	5.8	19	3.5	
Standard procedure	Yes	3225	86.2	371	91.4	1.251	84.2	488	80.4	< .001
	Right colectomy	1850	57.3	208	56.1	360	28.8	199	40.8	
	Left colectomy	1080	33.5	133	35.8	435	34.8	223	45.7	
	Anterior resection	295	9.2	30	8.1	456	36.4	66	13.5	
	No	517	13.8	35	8.6	235	15.8	119	19.6	
	Transverse colectomy	78	15.1	10	28.3	37	15.7	18	15.1	
	Splenic flexure colectomy	125	24.2	12	34.3	50	21.3	14	11.8	
	Hartmann reversal	84	16.3	4	11.5	63	26.8	12	10.1	
	(Sub) total colectomy	52	10.1	4	11.5	26	11.1	19	16.0	
	Other	178	34.3	5	14.4	59	25.1	56	47.0	
Anastomosis 1	Intracorporeal	2581	69.0	300	73.9	895	60.2	432	71.2	< .001
	Extracorporeal	1161	31.0	106	26.1	591	39.8	175	28.8	
Anastomosis 2	Stapled	3400	90.9	354	87.2	1.317	88.6	514	84.7	< .001
	Handsewn	342	9.1	52	12.8	169	11.4	93	15.3	
Anastomosis 3	End-to-end	1464	39.1	164	40.4	935	62.9	293	48.3	< .001
	Other shape	2278	60.9	242	59.6	551	37.1	314	51.7	
Operation length (minutes)	≤ 175	1965	52.5	236	58.1	628	42.3	364	60.0	< .001
	˃ 175	1777	47.5	170	41.9	858	57.7	243	40.0	
Hospital type	Met./Ac	2267	60.1	257	63.3	769	51.7	516	85.0	< .001
	Local/Regional	1475	39.4	149	36.7	717	48.3	91	15.0	
Unit type	Colorectal/oncologic	470	12.6	22	5.4	490	33.0	144	23.7	< .001
	General	3272	87.4	384	94.6	996	67.0	463	76.3	
Center volume	< 4 cases/month	887	23.7	136	33.5	449	30.2	221	36.4	< .001
	≥ 4 cases/month	2855	76.3	270	66.5	1.037	69.8	386	63.6	
Preoperative BT(s)	Yes	234	6.2	17	4.2	68	4.6	26	4.3	.023
	No	3508	93.8	389	95.8	1.418	95.4	581	95.7	
Intra/postoperative BT(s)	Yes	242	6.5	15	3.7	95	6.4	43	7.1	.141
	No	3500	93.5	391	96.3	1.391	93.6	564	92.9	
Overall ERAS adherence (%)	≤ 73.68	1271	34.0	88	21.7	1.108	74.6	209	34.4	< .001
	˃ 73.68	2471	66.0	318	78.3	378	25.4	398	65.6	
Nutritional screening		2780	74.3	301	74.1	914	61.5	410	67.6	
Prehabilitation		1730	46.2	228	56.2	276	18.6	183	30.2	
Counseling		2751	73.5	276	68.0	733	49.3	471	77.6	
Immune enhancing nutrition		1271	34.0	217	53.5	268	18.0	113	18.6	
Antithrombotic prophylaxis		3585	95.8	388	95.6	1.385	93.2	550	90.6	
Preoperative carbohydrates load		2505	66.9	256	63.1	517	34.8	326	53.7	
No preanesthesia		3265	87.3	293	77.2	867	58.3	448	73.8	
Standard anesthesia protocol		3188	85.2	396	97.5	934	62.9	584	96.2	
Normothermia		3572	95.5	398	98.0	1.211	81.5	576	94.9	
Goal-directed fluid therapy		3084	82.4	359	88.4	900	60.6	539	88.8	
PONV prophylaxis		3370	90.1	392	96.6	1.143	76.9	543	89.5	
Multimodal analgesia		3448	92.1	402	99.0	1.142	76.9	573	94.4	
No nasogastric tube		3376	90.2	391	96.3	1.127	75.8	491	80.9	
Minimally invasive surgery		3311	88.5	355	87.4	1.205	81.1	545	89.8	
No drains		1525	40.7	242	59.6	171	11.5	178	29.3	
Urinary catheter < 24–48 h		3096	82.7	380	93.6	832	56.0	484	79.7	
Early mobilization		2391	63.9	373	91.9	391	26.3	469	77.3	
Early oral feeding		2286	61.1	352	86.7	431	29.0	374	61.6	
Pre-discharge check		3275	87.5	345	85.0	848	57.1	503	82.9	

*NBP* no bowel preparation, *oA* oral antibiotics alone, *MBP* mechanical bowel preparation alone, *MoABP* mechanical bowel preparation and oral antibiotics, *ASA* American Society of Anesthesiologists, *MNA-SF* mini nutritional assessment–short form, *IBD* inflammatory bowel disease, Intracorporeal, anastomosis performed under visual control through the scope, Extracorporeal, anastomosis performed under direct visual control through an open access, *Met./Ac*., Metropolitan/Academic, *BT* blood transfusion, *ERAS*: Enhanced recovery after surgery, *PONV* postoperative nausea/vomiting, *p* chi square independence test with three degrees of freedom

### Outcomes

All the outcomes were calculated at 60 days after surgery. Any adverse event was recorded and graded [40, 41], as well as any reoperation, readmission, or death.

The primary endpoints were AL, defined according to the international consensus criteria [42], SSIs, according to the criteria of the Centers for Disease Control and Prevention/National Healthcare Safety Network (CDC/NHSN) [43], and overall morbidity (OM; any adverse event). The secondary endpoints were superficial and/or deep incisional surgical site infections (sdiSSIs), defined as specific complications including purulent drainage from superficial incisions, positive culture of fluid or tissue from superficial incisions, pain or tenderness, localized swelling, redness, heat, and/or infections involving deep fascial and muscle layers without fascial dehiscence; deep wound dehiscence; abdominal collection/abscess, defined as any intraperitoneal postoperative collection altering the normal postoperative course, requiring either medical, radiological, endoscopic, or surgical intervention [43]; major morbidity (any adverse event grade > II); reoperation (any unplanned operation); mortality (any death).

### Ethics

Both studies were conducted in accordance with the Declaration of Helsinki and guidelines for good clinical practice E6 (R2). All enrolled patients signed a consent to be included in the studies. The study protocols were approved by the ethics committee of the coordinating center (Marche Regional Ethics Committee (CERM) 2018/334 released on 11/28/2018 for iCral2 and 2020/192 released on 07/30/2020 for iCral3) and registered at ClinicalTrials.gov (NCT03771456 for iCral2 and NCT04397627 for iCral3). Subsequently, all other centers were authorized to participate in their local ethics committees. Both studies were approved for planned primary and any unplanned secondary analyses; therefore, no further authorization for the current analysis was requested. Individual participant-level anonymized datasets were made available upon reasonable request by contacting the study coordinator.

### Statistical analysis

Sample sizes were calculated and reported in the respective core papers [34, 35]. Events per variable guideline were followed [44]. There were no missing data in the database of 6241 patients. The target of estimands was represented by the average treatment effect in the true population of interest (ATT) answering the question “How would the average outcome(s) change if anyone receiving the reference treatment (NBP) had instead received another treatment?” A machine-learning technique, named the Generalized Boosted Model (GBM), was used to estimate the propensity score weights for the binary comparisons between the reference treatment and the other treatment arms. GBM estimation involves an iterative process with multiple regression trees to capture complex and nonlinear relationships between treatment assignment and the covariates without over-fitting the data [37]. The choice of GBM is due to a better balance of the features [37] and to an enhanced bias reduction [35] compared to other multinomial logistic regression models such as inverse probability weighting (IPWT). The analysis was performed using the “twang library” (Toolkit for Weighting and Analysis of Nonequivalent Groups,) of the software “R©” (Version 4.2.2, The R Foundation© for Statistical Computing, Vienna, Austria, 2022). As GBM works iteratively estimating the propensity scores according to the minimization of the distance of the weighted distributions of the covariates given the baseline treatment, balance comparisons have been estimated by performing 10,000 iterations and using the Kolmogorov–Smirnov (KS.mean) metrics with a threshold of 0.2 (a KS-mean difference less than 0.2 typically indicates a negligible difference between the means of the groups) [37]. The KS.mean was preferred based on the availability of a large sample size allowing comparison of the entire distribution rather than just of the mean.

Twenty covariates potentially affecting the four-treatments variable assignments [45] were included in the model (Fig. 1).

For the outcome analysis, weighted logistic regression models for both primary and secondary endpoints defined as dichotomous variables, according to the baseline treatment (NBP) and the other three treatment arms (oA, MBP, and MoABP), were estimated using the “svyglm library” (Survey General Linear Models) of the software “R^©^” (Version 4.2.2, The R Foundation^©^ for Statistical Computing, Vienna, Austria, 2022). The logistic regression models for the endpoints were adjusted considering the same 20 covariates used in the weight estimation, using a “doubly robust” estimation of the treatment effects [37]. Considering that the primary endpoints were not independent, having been selected based on available evidence [23], a Sidak–Bonferroni adjustment for multiple comparisons/outcomes was applied, calculating *α* = 0.012. Statistical significance, therefore, was accepted for *p* values < 0.012. All the instructions used with the software “R^©^” are available upon reasonable request to the study coordinator.

## Results

**Fig. 2:**
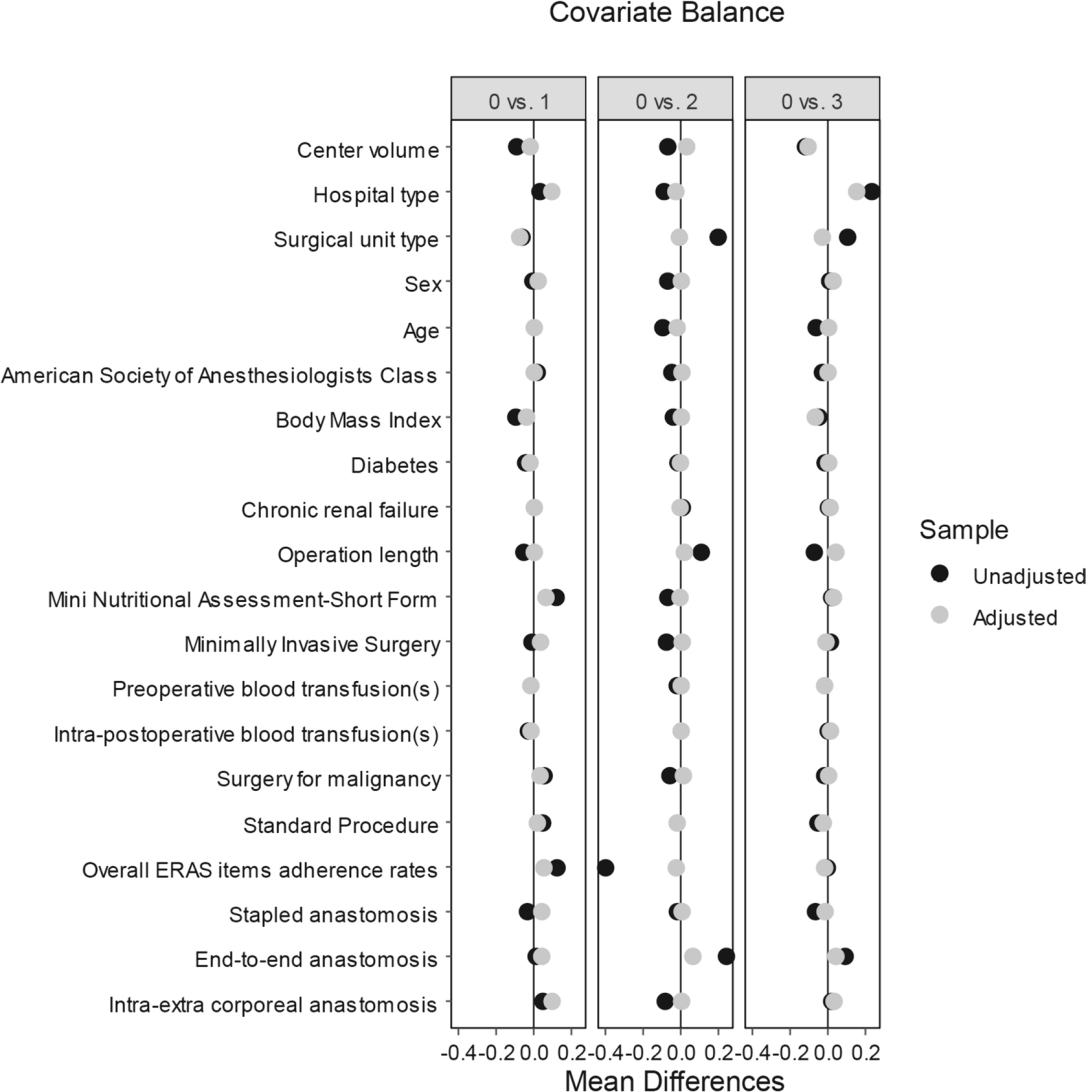
Love plot of covariates’ Kolmogorov–Smirnov mean differences before and after adjustment using a machine learning technique, comparing the reference treatment (no bowel preparation, named “0” in the figure) with the other 3 treatments (oral antibiotics alone, named “1”; mechanical bowel preparation alone, named “2”; mechanical bowel preparation and oral antibiotics, named “3”); ERAS, enhanced recovery after surgery

The population of 6241 patients included data deriving from 72 (92.3%) of the original 78 centers. NBP group included data deriving from 61 (84.7%), oA from 12 (16.7%), MBP from 52 (72.2%), and MoABP from 18 (25.0%) of the 72 centers. All the 20 covariates included in the model showed an optimal balance among treatment groups (Fig. 2).

**Fig. 3:**
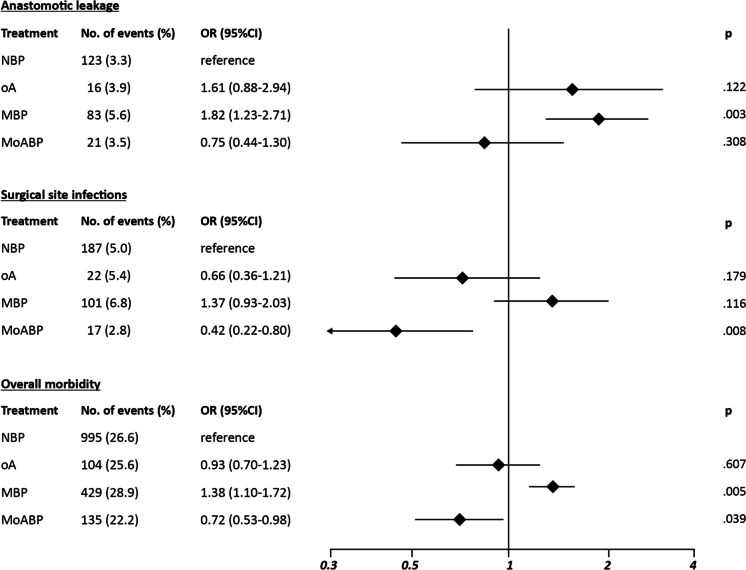
Multi-treatment weighted logistic regression analysis for primary endpoints (log scale); NBP, no bowel preparation; oA, oral antibiotics alone; MBP, mechanical bowel preparation alone; MoABP, mechanical bowel preparation and oral antibiotics

The multi-treatment weighted logistic regression analysis for primary endpoints (Fig. 3) showed the AL risk (3.3% after NBP) to be significantly higher after MBP (5.6%; OR 1.82; 95% CI 1.23–2.71; *p* = 0.003) and comparable after oA (3.9%) and MoABP (3.5%). The SSI risk (5.0% after NBP) was significantly lower after MoABP (2.8%; OR 0.42; 95% CI 0.22–0.80; *p* = 0.008) and comparable after oA (5.4%) and MBP (6.8%). The OM risk (26.6% after NBP) was significantly higher after MBP (28.9%; OR 1.38; 95% CI 1.10–1.72; *p* = 0.005), comparable after oA (25.6%) and MoABP (22.2%).

Concerning secondary endpoints (Table 3), no significant differences were recorded concerning the risk of deep wound dehiscence, abdominal collection/abscess, reoperation, and mortality. The risk of sdiSSI (3.3% after NBP) was significantly reduced after MoABP (1.7%; OR 0.29; 95% CI 0.14–0.60; *p* = 0.001), and the risk of major morbidity (5.3% after NBP) was significantly higher after oA (7.6%; OR 2.07; 95% CI 1.31–3.28; *p* = 0.002).

**Table 3:** Multi-treatment weighted logistic regression analysis for secondary endpoints

**Endpoint/treatment**	**NBP** (No. = 3742) OR (95% CI); p	**oA** (No. = 406) OR (95% CI); p	**MBP** (No. = 1486) OR (95% CI); p	**MoABP** (No. = 607) OR (95% CI); p
sdiSSIs	3.3% Reference	2.5% 0.67 (0.33–1.40); *p* = .285	4.9% 1.29 (0.81–2.07); *p* = .289	1.7% 0.29 (0.14–0.60); *p* = .001
Deep wound dehiscence	0.2% Reference	0.7% 3.08 (0.84–11.2); *p* = .089	0.3% 0.75 (0.19–2.96); *p* = .678	0.2% 0.50 (0.06–4.13); *p* = .521
Abdominal collection/abscess	1.7% Reference	0.7% 0.35 (0.08–1.51); *p* = .157	1.8% 1.53 (0.81–2.91); *p* = .190	1.0% 0.54 (0.15–1.88); *p* = .332
Major morbidity	5.3% Reference	7.6% 2.07 (1.31–3.28); *p* = .002	6.7% 1.04 (0.72–1.52); *p* = .825	4.9% 0.71 (0.46–1.12); *p* = .140
Reoperation	4.6% Reference	5.4% 1.48 (0.86–2.53); *p* = .158	6.2% 1.26 (0.86–1.85); *p* = .230	4.5% 0.76 (0.47–1.22); *p* = .250
Mortality	0.9% Reference	0.5% 0.86 (0.21–3.48); *p* = .833	1.0% 1.38 (0.61–3.11); *p* = .439	0.3% 0.62 (0.11–3.38); *p* = .578

*NBP* no bowel preparation, *oA* oral antibiotics alone, *MBP* mechanical bowel preparation alone, *MoABP* mechanical bowel preparation and oral antibiotics, *sdiSSIs* superficial and/or deep incisional surgical site infections

All the details regarding the multi-treatment machine learning adjusted comparisons are reported in the online supplemental material.

## Discussion

To the best of our knowledge, this is the first multi-treatment propensity score weighting analysis performed using the machine-learning weighted/adjusted regression model to assess different bowel preparation methods before elective colorectal surgery. When conclusive evidence from randomized trials is lacking or when researchers need to assess treatment effects based on real-life data, multiple treatments propensity score weighting analysis based on machine-learning methods performed on data from prospective observational studies offers an alternative approach for estimating treatment effects. The machine learning GBM model adopted in this study provides an improvement in bias reduction and external validity (not reducing the sample size analyzed) in comparison with propensity score-matching analyses between the ATT and the other treatments (three in the present study) and enhances bias reduction in comparison with IPWT [36, 37].

The main finding of the present analysis is that MoABP, compared to NBP, showed a significantly lower SSI risk, with no significant difference concerning the AL risk and a borderline reduction of the OM risk (Fig. 3). As the severity of complications comprised into OM rates may be skewed between groups and not captured by aggregate analysis, a detailed list of adverse events is reported in Table S4 in online supplemental material. This finding remained consistent with the analysis of secondary endpoints, with a significant reduction of the sdiSSI risk, without any significant difference regarding the risks of major morbidity, mortality, and reoperation (Table 3). Although the only available, though largely underpowered, randomized trial comparing NBP with MoABP [25] failed to detect any significant difference regarding SSI rates in the two arms, our results support the findings of the ACS-NSQIP retrospective series [9–13], the North American societies guidelines [14–16], and the most recent European guideline [31] towards the recommendation of MoABP in elective colorectal surgery. However, since both oA and MBP determine deep alterations of gut microbiota with possible impact on SSIs and AL rates [46], and considering that an optimal oral antibiotics administration schedule is far from being established in clinical practice (Table 1), the results of ongoing randomized trials comparing oA alone for colon resection [28] and MBP for rectal resections [22] with MoABP are eagerly awaited.

At the same time, no significant differences were recorded for all the primary endpoints concerning oA (Fig. 3), whereas it determined a significantly higher major morbidity risk (Table 3), possibly linked to a higher, though not significant, rate of major deep wound dehiscence, sdiSSIs, anastomotic leakage, and cardiac dysfunction events (Table S4 in online supplemental material).

Finally, MBP determined significantly higher AL and OM risks (Fig. 3), confirming the available evidence from randomized trials [1–4] and the findings of a recent propensity score-matched comparison of NBP vs. MBP alone performed on a more limited number of cases derived by the iCral database [5]. Considering that MBP alone was still used in nearly one-quarter of our cases, a de-implementation strategy or, according to the preference of some surgeons for a clean colon, a shift towards MoABP is highly advisable.

The main strength of the present study is represented by a large number of prospectively enrolled patients in a well-defined time-lapse in a large number of centers, treated by mini-invasive surgery in more than 80% of cases, representing a wide sample of surgical units performing colorectal resections in Italy. Although the multicenter nature of the data may be a definite source of clustering bias, it is undoubtedly representative of real-life clinical practice. Another strength is represented by its methodology (Fig. 1): (a) a reasoned selection of patients from the parent database was performed upon explicit criteria, limiting data imbalance; (b) the inclusion of 20 covariates into the model allowed to account for the potential clustering bias of multicenter data, for any confounder due to different perioperative pathways, to surgical approach and techniques, to blood transfusion-related morbidity [47], and to patient-related factors; (c) evaluation of the treatments effect through a weighted-adjusted regression model including the same 20 covariates [48]. Although the treatment groups were significantly unbalanced before GBM weighting (Table 2) concerning several well-known risk factors for the endpoints (i.e.,: age, sex, ASA class, nutritional status, minimally invasive surgery, type of resection, type and caseload of the recruiting center), the machine-learning generalized boosted model used in this study markedly improves bias reduction minimizing the distance of the weighted distributions of the 20 covariates (Fig. 2) compared to alternative methods such as IPWT [36, 37].

However, this study has several limitations, and its results should be interpreted with caution: (a) a relevant heterogeneity of oral antibiotic schedules (Table 1), as within and between previously published RCT and related meta-analyses [33]; (b) the exclusion criteria applied to the parent database (Fig. 1) practically excluded any resection performed for low rectal cancer, making the results not applicable to this subgroup of patients; (c) several aspects of health-acquired infections preventive bundle (preoperative whole-body bathing, hair removal, and skin decontamination) and single surgeon’s experience [49] were not measured in the parent studies; (d) finally, further bias from residual unknown factors and potential measurement errors by the participating investigators may have had an impact on the results.

## Conclusions

This multi-treatment machine learning analysis, despite the limitations mentioned above, showed that mechanical bowel preparation combined with oral antibiotics significantly reduced the SSI risk after elective colorectal surgery.

## Supplementary Information

Below is the link to the electronic supplementary material.

Supplementary file1 (DOCX 60 KB)

## Data Availability

All the datasets and all the instructions used with the software “R©” are available upon reasonable request to the corresponding author.
