# Supplementary material for: Bowel preparation for elective colorectal resection: multi-treatment machine learning analysis on 6241 cases from a prospective Italian cohort
Source: Int J Colorectal Dis. 2024 Apr 16;39(1):53. doi: 10.1007/s00384-024-04627-6 (PMC11021318; doi:10.1007/s00384-024-04627-6)
Supplement: Supplementary file 1 — Supplementary file1 (DOCX 60 KB) [file 384_2024_4627_MOESM1_ESM.docx]

**Bowel preparation for elective colorectal resection: multi-treatment machine-learning analysis on 6,241 cases from a prospective Italian cohort.**

Marco Catarci^1α^, MD, FACS, Stefano Guadagni^2, 3^, MD, Francesco Masedu^3^, PhD, Giacomo Ruffo^4^, MD, Massimo Giuseppe Viola^5^, MD, Felice Borghi^6^, MD, Gianluca Garulli^7^, MD, Felice Pirozzi^8^, MD, Paolo Delrio^9^, MD, Raffaele De Luca^10^, MD, Gianandrea Baldazzi^11^, MD, Marco Scatizzi^12^, MD, The Italian ColoRectal Anastomotic Leakage (iCral) study group*.

From the ^1^General Surgery Unit, Sandro Pertini Hospital, ASL Roma 2, Roma; ^2^General Surgery Unit, Università degli Studi dell’Aquila, L’Aquila; ^3^Department of Biotechnological and Applied Clinical Sciences, Università degli Studi dell’Aquila, L’Aquila; ^4^General Surgery Unit, IRCCS Sacro Cuore Don Calabria Hospital, Negrar di Valpolicella (VR); ^5^General Surgery Unit, Cardinale G. Panico Hospital, Tricase (LE); ^6^Oncologic Surgery Unit, Candiolo Cancer Institute, FPO-IRCCS, Candiolo (TO); ^7^General Surgery Unit, Infermi Hospital, Rimini; ^8^General Surgery Unit, ASL Napoli 2 Nord, Pozzuoli (NA); ^9^Colorectal Surgical Oncology, Istituto Nazionale per lo Studio e la Cura dei Tumori, “Fondazione Giovanni Pascale IRCCS-Italia”, Naples; ^10^Department of Surgical Oncology, IRCCS Istituto Tumori “Giovanni Paolo II”, Bari; ^11^General Surgery Unit, ASST Ovest Milanese, Legnano (MI); ^12^General Surgery Unit, Santa Maria Annunziata & Serristori Hospital, Florence; Italy.

**Corresponding Author:**

Stefano Guadagni, MD

Dipartimento di Scienze Cliniche Applicate e Biotecnologiche

Università degli Studi dell’Aquila, L’Aquila, Italy

Via Vetoio, snc; 67100 L’Aquila, Italy

Phone: +39 3339436171;

E-mail: [stefano.guadagni@univaq.it](mailto:stefano.guadagni@univaq.it); ORCID: 0000-0001-8525-084X

**Supplementary Online Materials - Index**

|  |  |
| --- | --- |
| Table S1 | *pag. 2* |
| Table S2 | *pag. 3* |
| Table S3 | *pag. 4* |
| Table S4 | *pag. 5* |
| STROBE Checklist | *pag. 6* |
|  |  |

**Table S1:** Binary comparison between NBP and oA groups.

| **Outcomes** | | **NBP** | |  | **oA** | | **Generalized Boosted Regression model** |
| --- | --- | --- | --- | --- | --- | --- | --- |
|  | **Pattern** | **No.** | **%** |  | **No.** | **%** | **OR (95%CI)** |
| s-d-SSI | Yes | 125 | 3.3 |  | 10 | 2.5 | 0.67 (0.33-1.40) p=.285 |
|  | No | 3,617 | 96.7 |  | 396 | 97.5 | Reference |
| Deep wound dehiscence | Yes | 8 | 0.2 |  | 3 | 0.7 | 3.08 (0.84-11.2) p=.089 |
|  | No | 3,734 | 99.8 |  | 403 | 99.3 | Reference |
| Abdominal collection/abscess | Yes | 63 | 1.7 |  | 3 | 0.7 | 0.35 (0.08-1.51) p=.157 |
|  | No | 3,679 | 98.3 |  | 403 | 99.3 | Reference |
| SSIs | Yes | 188 | 5.0 |  | 15 | 3.7 | 0.66 (0.36-1.21) p=.179 |
|  | No | 3,554 | 95.0 |  | 391 | 96.3 | Reference |
| Reoperation | Yes | 172 | 4.6 |  | 22 | 5.4 | 1.48 (0.86-2.53) p=.158 |
|  | No | 3,570 | 95.4 |  | 384 | 94.6 | Reference |
| Anastomotic leakage | Yes | 125 | 3.3 |  | 16 | 3.9 | 1.61 (0.88-2.94) p=.122 |
|  | No | 3,617 | 96.7 |  | 390 | 96.1 | Reference |
| Overall morbidity | Yes | 997 | 26.6 |  | 104 | 25.6 | 0.93 (0.70-1.23) p=.607 |
|  | No | 2,745 | 73.4 |  | 302 | 74.4 | Reference |
| Major morbidity | Yes | 198 | 5.3 |  | 31 | 7.6 | 2.07 (1.31-3.28) p=.002 |
|  | No | 3,544 | 94.7 |  | 375 | 92.4 | Reference |
| Mortality | Yes | 32 | 0.9 |  | 2 | 0.5 | 0.86 (0.21-3.48) p=.833 |
|  | No | 3,710 | 99.1 |  | 40 | 99.5 | Reference |

*NBP: no bowel preparation; oA: oral antibiotics alone;* *OR: odds ratio; 95%CI: 95% confidence interval; sdiSSIs: superficial and/or deep incisional surgical site infections; SSIs: Surgical Site Infections (sdiSSIs plus deep wound dehiscence plus abdominal collection/abscess).*

**Table S2:** Binary comparison between NBP and MBP groups.

| **Outcomes** | | **NBP** | |  | **MBP** | | **Generalized Boosted Regression model** |
| --- | --- | --- | --- | --- | --- | --- | --- |
|  | **Pattern** | **No.** | **%** |  | **No.** | **%** | **OR (95%CI)** |
| sdiSSIs | Yes | 125 | 3.3 |  | 73 | 4.9 | 1.29 (0.81-2.07) p=.289 |
|  | No | 3,617 | 96.7 |  | 1,413 | 95.1 | Reference |
| Deep wound dehiscence | Yes | 8 | 0.2 |  | 5 | 0.3 | 0.75 (0.19-2.96) p=.678 |
|  | No | 3,734 | 99.8 |  | 1,481 | 99.7 | Reference |
| Abdominal collection/abscess | Yes | 63 | 1.7 |  | 26 | 1.8 | 1.53 (0.81-2.91) p=.190 |
|  | No | 3,679 | 98.3 |  | 1,460 | 98.2 | Reference |
| SSIs | Yes | 188 | 5.0 |  | 101 | 6.8 | 1.37 (0.93-2.03) p=.116 |
|  | No | 3,554 | 95.0 |  | 1,385 | 93.2 | Reference |
| Reoperation | Yes | 172 | 4.6 |  | 92 | 6.2 | 1.26 (0.86-1.85) p=.230 |
|  | No | 3,570 | 95.4 |  | 1,394 | 93.8 | Reference |
| Anastomotic leakage | Yes | 125 | 3.3 |  | 83 | 5.6 | 1.82 (1.23-2.71) p=.003 |
|  | No | 3,617 | 96.7 |  | 1,403 | 94.4 | Reference |
| Overall morbidity | Yes | 997 | 26.6 |  | 430 | 28.9 | 1.38 (1.10-1.72) p=.005 |
|  | No | 2,745 | 73.4 |  | 1,056 | 71.1 | Reference |
| Major morbidity | Yes | 198 | 5.3 |  | 100 | 6.7 | 1.04 (0.72-1.52) p=.825 |
|  | No | 3,544 | 94.7 |  | 1,386 | 93.3 | Reference |
| Mortality | Yes | 32 | 0.9 |  | 15 | 1.0 | 1.38 (0.61-3.11) p=.439 |
|  | No | 3,710 | 99.1 |  | 1,471 | 99.0 | Reference |

*NBP: no bowel preparation; MBP: mechanical bowel preparation alone; OR: odds ratio; 95%CI: 95% confidence interval; sdiSSIs: superficial and/or deep incisional surgical site infections; SSIs: Surgical Site Infections (sdiSSIs plus deep wound dehiscence plus abdominal collection/abscess).*

**Table S3:** Binary comparison between NBP and MoABP groups.

| **Outcomes** | | **NBP** | |  | **MoABP** | | **Generalized Boosted Regression model** |
| --- | --- | --- | --- | --- | --- | --- | --- |
|  | **Pattern** | **No.** | **%** | **vs** | **No.** | **%** | **OR (95%CI)** |
| sdiSSIs | Yes | 125 | 3.3 |  | 10 | 1.7 | 0.29 (0.14-0.60) p=.001 |
|  | No | 3,617 | 96.7 |  | 597 | 98.3 | Reference |
| Deep wound dehiscence | Yes | 8 | 0.2 |  | 1 | 0.2 | 0.50 (0.06-4.13) p=.521 |
|  | No | 3,734 | 99.8 |  | 606 | 99.8 | Reference |
| Abdominal collection/abscess | Yes | 63 | 1.7 |  | 6 | 1.0 | 0.54 (0.15-1.88) p=.332 |
|  | No | 3,679 | 98.3 |  | 601 | 99.0 | Reference |
| SSIs | Yes | 188 | 5.0 |  | 17 | 2.8 | 0.42 (0.22-0.80) p=.008 |
|  | No | 3,554 | 95.0 |  | 590 | 97.2 | Reference |
| Reoperation | Yes | 172 | 4.6 |  | 27 | 4.5 | 0.76 (0.47-1.22) p=.250 |
|  | No | 3,570 | 95.4 |  | 580 | 95.5 | Reference |
| Anastomotic leakage | Yes | 125 | 3.3 |  | 21 | 3.5 | 0.75 (0.44-1.30) p=.308 |
|  | No | 3,617 | 96.7 |  | 586 | 96.5 | Reference |
| Overall morbidity | Yes | 997 | 26.6 |  | 135 | 22.2 | 0.72 (0.53-0.98) p=.039 |
|  | No | 2,745 | 73.4 |  | 472 | 77.8 | Reference |
| Major morbidity | Yes | 198 | 5.3 |  | 30 | 4.9 | 0.71 (0.46-1.12) p=.140 |
|  | No | 3,544 | 94.7 |  | 577 | 95.1 | Reference |
| Mortality | Yes | 32 | 0.9 |  | 2 | 0.3 | 0.62 (0.11-3.38) p=.578 |
|  | No | 3,710 | 99.1 |  | 605 | 99.7 | Reference |

*NBP: no bowel preparation; MoABP: mechanical bowel preparation and oral antibiotics; OR: odds ratio; 95%CI: 95% confidence interval; sdiSSIs: superficial and/or deep incisional surgical site infections; SSIs: Surgical Site Infections (sdiSSIs plus deep wound dehiscence plus abdominal collection/abscess).*

**Table S4**: Adverse events contributing to overall morbidity (OM, any adverse event) and major morbidity (MM, any adverse event grade > II) after elective colorectal surgery in 6,241 patients.

| **Adverse event** | **NBP**  **(No.= 3,742)** | | | **oA**  **(No.= 406)** | | | **MBP**  **(No.= 1,486)** | | | **MoABP**  **(No.= 607)** | | |
| --- | --- | --- | --- | --- | --- | --- | --- | --- | --- | --- | --- | --- |
|  | OM (%) | MM (%) | OM (%) | | MM (%) | OM (%) | | MM (%) | OM (%) | | MM (%) |  |
| Anastomotic leakage | 3.3 | 2.7 | 3.9 | | 3.7 | 5.6 | | 4.7 | 3.5 | | 3.0 |  |
| sdiSSIs | 3.3 | 0.2 | 2.5 | | 0.7 | 4.9 | | 0.5 | 1.7 | | 0.0 |  |
| Deep wound dehiscence | 0.2 | 0.1 | 0.7 | | 0.5 | 0.3 | | 0.0 | 0.2 | | 0.2 |  |
| Abdominal collection/abscess | 1.7 | 1.0 | 0.7 | | 0.2 | 1.8 | | 1.0 | 1.0 | | 0.2 |  |
| Small bowel obstruction | 1.3 | 0.9 | 1.5 | | 1.0 | 0.9 | | 0.6 | 2.3 | | 1.5 |  |
| Anastomotic bleeding | 2.0 | 0.5 | 2.5 | | 0.2 | 1.3 | | 0.4 | 1.6 | | 0.7 |  |
| Abdominal bleeding | 1.1 | 0.7 | 0.2 | | 0.2 | 1.1 | | 0.7 | 1.2 | | 0.3 |  |
| Small bowel perforation | 0.3 | 0.3 | 0.0 | | 0.0 | 0.1 | | 0.1 | 0.3 | | 0.3 |  |
| Trocar/wound site bleeding | 0.4 | 0.1 | 0.2 | | 0.0 | 0.3 | | 0.0 | 0.5 | | 0.0 |  |
| Anemia | 5.1 | 0.1 | 3.4 | | 0.2 | 4.8 | | 0.1 | 3.8 | | 0.0 |  |
| Paralytic ileus | 4.4 | 0.1 | 3.7 | | 0.0 | 4.0 | | 0.0 | 2.8 | | 0.0 |  |
| Fever | 3.4 | 0.1 | 3.2 | | 0.0 | 4.3 | | 0.1 | 2.5 | | 0.0 |  |
| DVT/pulmonary embolism | 0.3 | 0.1 | 0.2 | | 0.0 | 0.3 | | 0.1 | 0.3 | | 0.2 |  |
| Neurologic | 0.6 | 0.0 | 0.2 | | 0.0 | 0.4 | | 0.0 | 0.3 | | 0.2 |  |
| Pneumonia and pulmonary failure | 1.5 | 0.4 | 3.9 | | 0.5 | 2.0 | | 0.7 | 1.5 | | 0.2 |  |
| Urinary retention | 1.3 | 0.0 | 0.5 | | 0.0 | 1.3 | | 0.0 | 0.7 | | 0.0 |  |
| Urinary tract infection | 0.2 | 0.0 | 0.5 | | 0.2 | 0.5 | | 0.0 | 0.3 | | 0.0 |  |
| Acute renal failure | 0.7 | 0.1 | 1.0 | | 0.0 | 0.7 | | 0.1 | 0.7 | | 0.2 |  |
| Acute mesenteric ischemia | 0.0 | 0.0 | 0.0 | | 0.0 | 0.2 | | 0.2 | 0.0 | | 0.0 |  |
| Acute peptic ulcer/erosive gastritis | 0.1 | 0.1 | 0.0 | | 0.0 | 0.1 | | 0.1 | 0.0 | | 0.0 |  |
| Cardiac dysfunction and failure | 1.4 | 0.3 | 1.0 | | 0.5 | 1.5 | | 0.3 | 0.8 | | 0.3 |  |
| Other | 5.3 | 0.8 | 4.2 | | 0.5 | 4.4 | | 0.8 | 4.6 | | 0.3 |  |

*NBP: no bowel preparation; oA: oral antibiotics; MBP: mechanical bowel preparation; MoABP: mechanical bowel preparation and oral antibiotics; OM: overall morbidity; MM: major morbidity; sdiSSIs: Superficial and/or deep incisional surgical site infections; DVT: deep venous thrombosis..*

STROBE Statement—Checklist of items that should be included in reports of ***cohort studies***

|  | Item No | Recommendation | Page No |
| --- | --- | --- | --- |
| **Title and abstract** | 1 | (*a*) Indicate the study’s design with a commonly used term in the title or the abstract |  |
|  |  | (*b*) Provide in the abstract an informative and balanced summary of what was done and what was found | 1 |
| Introduction | | | |
| Background/rationale | 2 | Explain the scientific background and rationale for the investigation being reported | 3 |
| Objectives | 3 | State specific objectives, including any prespecified hypotheses | 4 |
| Methods | | | |
| Study design | 4 | Present key elements of study design early in the paper | 4 |
| Setting | 5 | Describe the setting, locations, and relevant dates, including periods of recruitment, exposure, follow-up, and data collection | 4 |
| Participants | 6 | (*a*) Give the eligibility criteria, and the sources and methods of selection of participants. Describe methods of follow-up | 4-5 |
|  |  | (*b*) For matched studies, give matching criteria and number of exposed and unexposed |  |
| Variables | 7 | Clearly define all outcomes, exposures, predictors, potential confounders, and effect modifiers. Give diagnostic criteria, if applicable | 5 |
| Data sources/ measurement | 8* | For each variable of interest, give sources of data and details of methods of assessment (measurement). Describe comparability of assessment methods if there is more than one group | 4-5 |
| Bias | 9 | Describe any efforts to address potential sources of bias | 5 |
| Study size | 10 | Explain how the study size was arrived at | 5 |
| Quantitative variables | 11 | Explain how quantitative variables were handled in the analyses. If applicable, describe which groupings were chosen and why | 5 |
| Statistical methods | 12 | (*a*) Describe all statistical methods, including those used to control for confounding | 6-7 |
|  |  | (*b*) Describe any methods used to examine subgroups and interactions |  |
|  |  | (*c*) Explain how missing data were addressed |  |
|  |  | (*d*) If applicable, explain how loss to follow-up was addressed |  |
|  |  | (*e*) Describe any sensitivity analyses |  |
| Results | | |  |
| Participants | 13* | (a) Report numbers of individuals at each stage of study—eg numbers potentially eligible, examined for eligibility, confirmed eligible, included in the study, completing follow-up, and analysed | 7 |
|  |  | (b) Give reasons for non-participation at each stage |  |
|  |  | (c) Consider use of a flow diagram |  |
| Descriptive data | 14* | (a) Give characteristics of study participants (eg demographic, clinical, social) and information on exposures and potential confounders | 7 |
|  |  | (b) Indicate number of participants with missing data for each variable of interest |  |
|  |  | (c) Summarise follow-up time (eg, average and total amount) |  |
| Outcome data | 15* | Report numbers of outcome events or summary measures over time | 7 |

| Main results | 16 | (*a*) Give unadjusted estimates and, if applicable, confounder-adjusted estimates and their precision (eg, 95% confidence interval). Make clear which confounders were adjusted for and why they were included | 7-8 |
| --- | --- | --- | --- |
|  |  | (*b*) Report category boundaries when continuous variables were categorized |  |
|  |  | (*c*) If relevant, consider translating estimates of relative risk into absolute risk for a meaningful time period |  |
| Other analyses | 17 | Report other analyses done—eg analyses of subgroups and interactions, and sensitivity analyses | n.a. |
| Discussion | | | |
| Key results | 18 | Summarise key results with reference to study objectives | 8 |
| Limitations | 19 | Discuss limitations of the study, taking into account sources of potential bias or imprecision. Discuss both direction and magnitude of any potential bias | 8-9 |
| Interpretation | 20 | Give a cautious overall interpretation of results considering objectives, limitations, multiplicity of analyses, results from similar studies, and other relevant evidence | 8-9 |
| Generalisability | 21 | Discuss the generalisability (external validity) of the study results | 8-9 |
| Other information | | | |
| Funding | 22 | Give the source of funding and the role of the funders for the present study and, if applicable, for the original study on which the present article is based | 2 |
